# Hospice Care Inequities in Individuals With Alzheimer's Disease and Related Dementias

**DOI:** 10.1093/geroni/igab046.1277

**Published:** 2021-12-17

**Authors:** Abraham Brody, Leah Estrada, Aditi Durga, Shih-Yin Lin, Ariel Ford

**Affiliations:** 1 NYU Hartford Institute for Geriatric Nursing, New York, New York, United States; 2 Columbia University School of Nursing, New York, New York, United States; 3 NYU Rory Meyers College of Nursing, New York, New York, United States; 4 New York University, New York, New York, United States

## Abstract

Despite known benefits of hospice, inequities exist. Using data from a multi-site pragmatic trial in a representative groups of hospices, we examined inequities in length of stay (LOS) and general inpatient use (GIU) for 12,153 patients with dementia (primary and secondary diagnosis) using descriptive statistics and association tests. There were significant associations between race/ethnicity and GIU and LOS (p< 0.001). In those with primary diagnosis of dementia, Asian (31%) and Black/AA (24%) individuals had significantly greater utilization of GIU than Hispanic (19%) and white individuals (21%). Greater inequities were found in those with a secondary diagnosis. LOS amongst Asians were shortest with 78% having an LOS ≦14 vs 50-59% in other groups. Differences in long-stay >60 days (7%) vs 14-22% in other groups were found. There were similar differences examining by primary vs. secondary diagnosis. These inequities point to cultural and systems factors that require further study and intervention.

